# Characters of KRT80 and its roles in neoplasms diseases

**DOI:** 10.1002/cam4.6040

**Published:** 2023-05-21

**Authors:** Xin‐Yuan Wei, Jie Zhao, Hao‐Bin Tong, Shang‐Jie Cheng, Na He, Fei‐Xue Song

**Affiliations:** ^1^ Lanzhou University Second Hospital Lanzhou Gansu China; ^2^ Department of oncology Lanzhou University Second Hospital Lanzhou Gansu China

**Keywords:** cancers, intermediate filaments, keratin, KRT80, mechanism, neoplasms

## Abstract

**Background:**

KRT80 is a human epithelial intermediate filament type II gene; its expression product is a component of intracellular intermediate filaments (IFs) and is involved in the assembly of the cytoskeleton. There is evidence that IFs form a dense network mainly in the perinuclear area, but they can also reach the cortex. They are essential for mechanical cushioning of cells, organelle positioning, cell apoptosis, migration, adhesion, and interactions with other cytoskeletal components. Humans possess 54 functional keratin genes, and KRT80 is one of the more unique genes. It is widely expressed in almost all epithelial cells, although it is structurally more similar to type II hair keratins than to type II epithelial keratins.

**Aim:**

In this review, we summarize the basic facts about the keratin family and KRT80, the essential role of KRT80 in neoplasms, and its potential as a therapeutic target. We hope that this review will inspire researchers to at least partially focus on this area.

**Result:**

In many neoplastic diseases, the high expression status of KRT80 and its role in regulating the biological functions of cancer cells have been well established. KRT80 can effectively enhance the proliferation, invasiveness and migration of cancer cells. However, the effects of KRT80 on prognosis and clinically relevant indices in patients with various cancers have not been extensively studied, and even opposite conclusions have been reached in different studies of the same cancer. Based on this, we should add more clinically relevant studies to clarify the prospect of clinical application of KRT80. Many researchers have made great progress in studying the mechanism of action of KRT80. However, their studies should be extended to more cancers to find common regulators and signaling pathways of KRT80 in different cancers. KRT80 may have far‐reaching effects on the human body, and this marker may play a crucial role in the function of cancer cells and the prognosis of cancer patients, so it has a promising future in the field of neoplasms.

**Conclusion:**

In neoplastic diseases, KRT80 is overexpressed in many cancers and plays an essential role in promoting proliferation, migration, invasiveness and poor prognosis. The mechanisms of KRT80 functions in cancer have been partially elucidated, suggesting that KRT80 is a potentially useful cancer therapeutic target. However, more systematic, in‐depth and comprehensive studies are still needed in this field.

## 1 INTRODUCTION

There are many different structures within human cells, but the cytoskeleton is one of the most complex and diverse. The cytoskeleton plays an irreplaceable role in maintaining cell morphology, division, differentiation, motility, adhesion, and other processes. All these functions depend on a complex network of three classical cytoskeletal filaments: actin, microtubules, and intermediate filaments (IFs). In many diseases, including neoplasms, diseased cells achieve specific cell biological functions by altering the composition and structure of their cytoskeleton. Although we currently have little understanding of the principles and mechanisms by which cells modify the components of their cytoskeleton to alter its structure, this does not prevent us from investigating the relationship between changes in specific cytoskeletal components and disease development and regression. In fact, these studies will progressively deepen our understanding of the cytoskeleton.

IFs get their name from the fact that they have a diameter of 10 nm, intermediate between actin (6 nm) and microtubules (25 nm).^1^ In human cells, IFs form a dense network, the form and structure of which depends on the type of intermediate filament, and are mostly located in the perinuclear area, but can also reach the cortex.^2^, ^3^ In the vicinity of the cortex, IFs are involved in maintaining cell and tissue adhesion by interacting with adhesion junctions, desmosomes, and hemidesmosomes.^4^, ^5^, ^6^, ^7^ By interacting with these structures, IFs physically connect to the nuclear and plasma membranes, forming scaffolds and organizing the location of organelles such as mitochondria and the Golgi apparatus.^8^, ^9^, ^10^ Due to their ability to form network structures and to anchor organelles, IFs are generally considered to provide a mechanical cushion for the cell.^11^, ^12^, ^13^ They are also highly dynamic and rapidly changing cytoskeletal components with multiple functions, including roles in cell apoptosis, migration, adhesion, and interactions with other cytoskeletal components.^2^ To achieve these functions, IFs undergo many types of post‐translational modifications, such as ubiquitination and SUMOylation,^14^, ^15^, ^16^ which regulate their organization and assembly.

KRT80 is a human IF type II epithelial keratin gene involved in the formation of IF heterodimers in various epithelial cells. In recent years, numerous studies have shown that KRT80 regulates biological functions and patient prognosis in neoplasms. KRT80 is overexpressed in many neoplasms and plays an essential role in promoting cell proliferation, migration and invasiveness, and is associated with poor prognosis in cancer patients. The mechanisms of the roles KRT80 plays in cancer have been partially elucidated, but the molecular mechanisms underlying these processes need to be further clarified. In this review, we summarize the basic facts about the keratin family and KRT80, the essential role of KRT80 in neoplasms, and its potential as a therapeutic target. We hope that this review will inspire researchers to at least partially focus on this area.

## 2 MEMBERS OF THE KERATIN FAMILY

The keratin IF network is an essential part of the cytoskeleton in the cytoplasm of most eukaryotic cells. In total, there are about 70 genes that encode different IF proteins.^17^ They can be divided into five categories based on their structure and sequence homology; the first four represent cytoplasmic intermediate filaments, while type V are nuclear filaments called lamins. Type I and II are acidic and basic keratins that form a heterogeneous polymer composed of a mixture of 54 different type I and II keratins, including the KRT80 protein; type III intermediate filaments are homopolymers of vimentin, desmin, peripherin, or glial fibrillary acidic protein; type IV intermediate filaments contain three neurofilament heteropolymers, including internexin, synemin, and nestin, which are mainly expressed in cells of the nervous system.^2^

Genome analyses have already shown that humans possess 54 functional keratin genes, which can be divided into Type I and Type II based on their amino acid composition.^17^ Twenty eight Type I keratins are acidic keratins with genes located on chromosome 17q21.2.^18^ In contrast, 26 type II keratins are basic to neutral keratins with genes located on chromosome 12q13.13, except for KRT18, which is located in the type II keratin gene domain.^19^ Human keratins can also be classified according to their major sites of expression as epithelial keratins, typical of epithelial tissues, or hair keratins, mainly found in hair and nails.^17^ In a new consensus nomenclature for mammalian keratins that combines the two classification methods, human functional keratins have been classified into four types: 17 type I epithelial keratins (KRT9‐28), 11 type I hair keratins (KRT 31–40), 20 type II epithelial keratins (KRT1‐8 and 71–80), and 6 type II hair keratins (KRT81‐86) (Table 1).^17^

**TABLE 1:** Classification of human keratins/genes family.

Human IF type I keratins (gene symbols)	Chromosome localization	Human IF type II keratins (gene symbols)	Chromosome localization
*Human epithelial keratins*			
K9(*KRT9*)	17q21.2	K1(*KRT1*)	chr12q13.13
K10(*KRT10*)	17q21.2	K2(*KRT2*)	chr12q13.13
K12(*KRT12*)	17q21.2	K3(*KRT3*)	chr12q13.13
K13(*KRT13*)	17q21.2	K4(*KRT4*)	chr12q13.13
K14(*KRT14*)	17q21.2	K5(*KRT5*)	chr12q13.13
K15(*KRT15*)	17q21.2	K6a(*KRT6A*)	chr12q13.13
K16(*KRT16*)	17q21.2	K6b(*KRT6B*)	chr12q13.13
K17(*KRT17*)	17q21.2	K6cc(*KRT6C*)	chr12q13.13
K18(*KRT18*)	chr12q13.13	K7(*KRT7*)	chr12q13.13
K19(*KRT19*)	17q21.2	K8(*KRT8*)	chr12q13.13
K20(*KRT20*)	17q21.2	K71(*KRT71*)	chr12q13.13
K23(*KRT23*)	17q21.2	K72(*KRT72*)	chr12q13.13
K24(*KRT24*)	17q21.2	K73(*KRT73*)	chr12q13.13
K25(*KRT25*)	17q21.2	K74(*KRT74*)	chr12q13.13
K26(*KRT26*)	17q21.2	K75(*KRT75*)	chr12q13.13
K27(*KRT27*)	17q21.2	K76(*KRT76*)	chr12q13.13
K28(*KRT28*)	17q21.2	K77(*KRT77*)	chr12q13.13
		K78(*KRT78*)	chr12q13.13
		K79(*KRT79*)	chr12q13.13
		K80(*KRT80*)	chr12q13.13
*Human hair keratins*			
K31(*KRT31*)	17q21.2	K81(*KRT81*)	chr12q13.13
K32(*KRT32*)	17q21.2	K82(*KRT82*)	chr12q13.13
K33a(*KRT33A*)	17q21.2	K83(*KRT83*)	chr12q13.13
K33b(*KRT33B*)	17q21.2	K84(*KRT84*)	chr12q13.13
K34(*KRT34*)	17q21.2	K85(*KRT85*)	chr12q13.13
K35(*KRT35*)	17q21.2	K86(*KRT86*)	chr12q13.13
K36(*KRT36*)	17q21.2		
K37(*KRT37*)	17q21.2		
K38(*KRT38*)	17q21.2		
K39(*KRT39*)	17q21.2		
K40(*KRT40*)	17q21.2		

## 3 CHARACTERS OF *KRT80*

KRT80 is a human IF type II epithelial keratin gene. The KRT80 gene is located on chromosome 12q13.13, the terminal centromere of the human type II keratin gene domain.^20^ The KRT80 keratin chain contains 452 amino acids and has a molecular mass of 50.5 kDa and an isoelectric pH of 5.0.^20^ In addition, the KRT80 gene has been found to have an alternative splice variant, KRT80.1, which differs from KRT80 in the last exon of the KRT80 gene,^21^ but the parameters of this protein have not been specified.

In the human body, KRT80 has been demonstrated to be widely expressed in almost all epithelial cells, including stratified keratinizing and non‐keratinizing, hard keratinizing and non‐stratified tissues, and cultured epithelial cells. Accordingly, KRT80 can bind to more than 20 different type II keratins to form an IF heterodimer in various epithelial cells.^21^ More interestingly, KRT80‐containing IFs were localized at the cell margins near the desmosome plaques in the early stages of differentiation and were dispersed throughout the cytoplasm in terminally differentiated cells, distinguishing KRT80 from other typical keratins.^21^

## 4 STUDY OF *KRT80* IN NEOPLASTIC DISEASES

Due to the widespread expression of KRT80 in epithelial cells and its many unique properties, researchers have been investigating its role in disease, particularly in neoplasms, including its effects on the biological functions of cancer cells, mechanisms, and patient prognosis (Table 2). To date, many related studies are still being conducted, and it is believed that more findings will be published in the future.

**TABLE 2:** Summary of the study of KRT80 in neoplastic diseases.

Tumor type	Upstream regulators/pathways	Downstream regulators/pathways	Result	Reference
ESCC	miR‐143‐3p	N/A	Enhance proliferation, invasiveness, and migration.	Wada, Goto^22^
GC	circPIP5K1A/miR‐671‐5p	PI3K/AKT	Enhance proliferation, invasiveness, and migration.	Li, Liu^23^
GC	OTUB2	PI3K/AKT	Enhance proliferation.	Ouyang, Zeng^24^
GC	N/A	ANXA10	Enhance proliferation and invasiveness.	Ishikawa, Sakamoto^25^
CRC	PRKDC; PPP1CA	PI3K/AKT	Enhance proliferation, invasiveness, migration, and viability; associated with poorer DFS and OS.	Li, Liu,^23^ Lin, Fan^26^
CRC	CTNNB1	Wnt; exosomes	Not significantly associated with patient outcomes.	Ma, Wang,^27^ Wang, Huang^28^
NSCLC	N/A	N/A	Not significantly associated with clinicopathologic factors.	夏雨婷^29^
NSCLC	N/A	N/A	Associated with poorer prognosis.	Sanada, Seki^30^
BC	SREBP1	N/A	Induce cytoskeletal changes and lamellipodia, endocrine resistance; enhance invasiveness.	Nguyen, Barozzi,^31^ Perone, Farrugia^32^
OC	miR‐206/ETS1	MEK/ERK	Enhance proliferation, invasiveness, migration; associated with later FIGO stage and higher rate of lymph node metastasis.	Liu, Wang^33^
FTC	N/A	N/A	Influenced by short‐term microgravity.	Ulbrich, Pietsch^34^
ccRCC	N/A	N/A	Linked to metabolism, immune infiltration, and immune checkpoints.	Chen, Liang^35^
PCCs	N/A	N/A	Enhance proliferation, invasiveness, and migration.	Wang, Huang^28^
SKCM	TCONS_00049140	N/A	Involved in tumor suppression in mouse melanoma.	Ji, Fan^36^

### 4.1 KRT80 and esophageal squamous cell carcinoma (ESCC)

Using an immunostaining assay, Wada et al found that KRT80 expression is rarely observed in normal epithelium, but is over‐expressed in ESCC clinical specimens. Using siKRT80 to knock down KRT80 in ESCC cells, they confirmed the oncogenic function of KRT80 in ESCC cells. The results showed that the proliferation, migration, and invasiveness of ESCC cells were significantly reduced by suppressing KRT80 expression; that is, the expression of KRT80 enhanced the malignancy of ESCC cells.^22^ In addition, the overexpression of KRT80 is likely caused by the downregulation of miR‐143‐3p in ESCC cells, as miR‐143‐3p was found to target KRT80 and reduce it when miR‐143‐3p was transfected into ESCC cells.^22^

In this study, the role of KRT80 in the biological function of ESCC was investigated in detail, and its possible regulatory pathways and mechanisms were partially revealed. However, whether the effects of KRT80 may adversely affect the prognosis of patients and its detailed upstream and downstream regulatory information remain to be further explored.

### 4.2 KRT80 and gastric cancer (GC)

**FIGURE 1:**
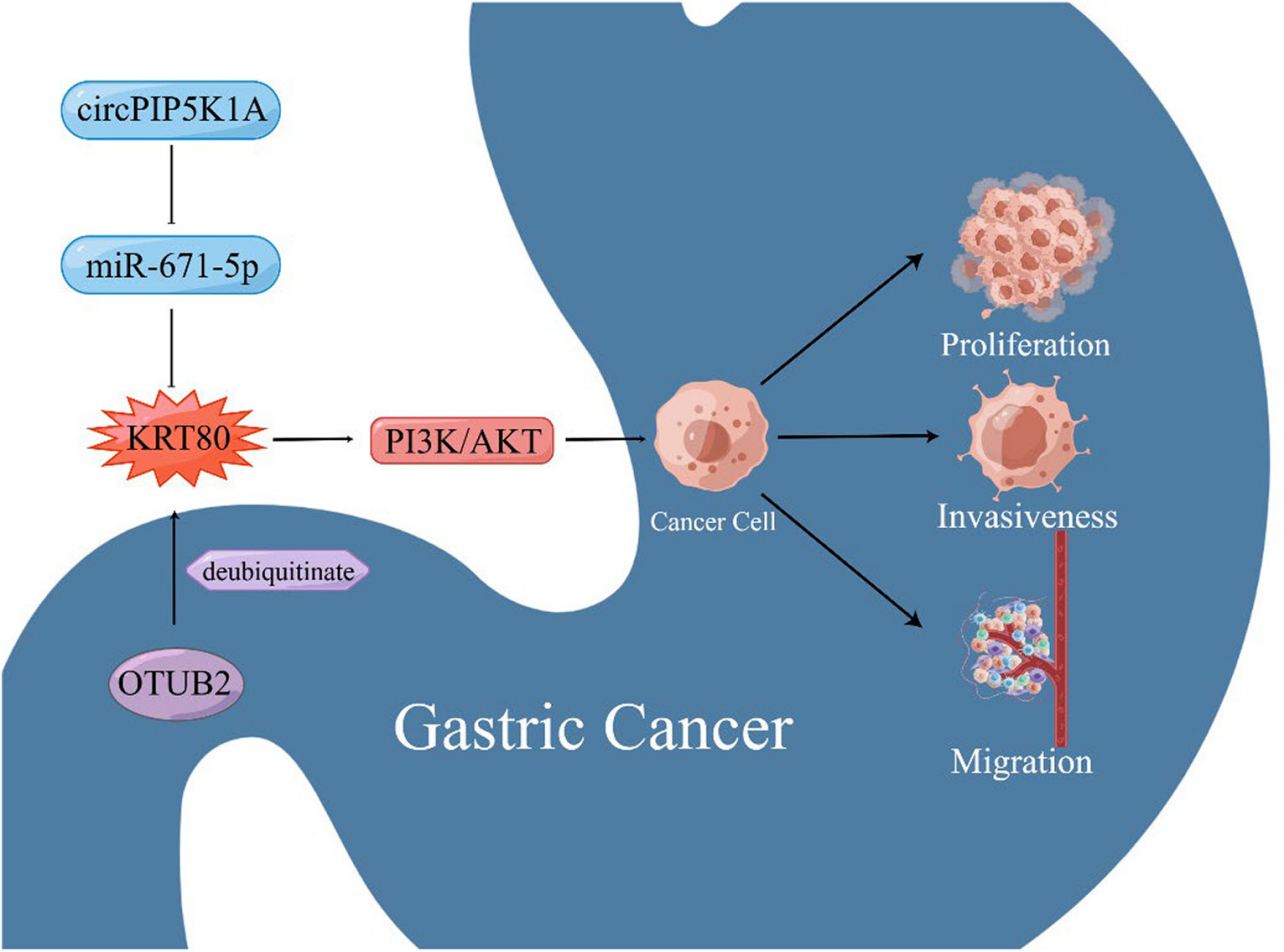
The roles of KRT80 in gastric cancer.

Using a PCR assay, Song et al found that there was a significant increase in KRT80 expression in GC tissues compared to normal tissues. Using siKRT80 to downregulate KRT80 in MKN45 and AGS cells, they investigated the role of KRT80 in GC. Based on colony formation and transwell assays, the results showed that KRT80 deficiency inhibited the proliferation, invasiveness and migration abilities of GC cells. More importantly, the role of KRT80 was mediated through the circPIP5K1A‐miR‐671‐5p‐KRT80 axis and the PI3K/AKT pathway in GC cells (Figure 1). The researchers found that circPIP5K1A expression was upregulated and miR‐671‐5p expression was downregulated in GC tissues compared to normal tissues. In addition, suppression of circPIP5K1A can enhance the expression of miR‐671‐5p, and other relevant assays all support the conclusion that circPIP5K1A functions as a ceRNA and sponge miR‐671‐5p in GC cells. They also found that KRT80 expression was significantly reduced by miR‐671‐5p mimics and vice versa in a PCR assay. Furthermore, other relevant assays all support the conclusion that KRT80 is directly negatively regulated by miR‐671‐5p. Li et al showed that the PI3K/AKT pathway is activated by KRT80 through its interaction with PRKDC,^23^ and this role was also demonstrated by a western blot assay in GC. Regulation of KRT80 by circPIP5K1A, as a whole, was demonstrated using PCR, colony formation, transwell and other relevant assays.^37^ In the study of Fangqing et al, overexpression of KRT80 was similarly proven in the GC tissues of 20 GC patients, and KRT80 expression levels in GC tissues were correlated with lymph node metastasis in patients.^38^

In the most recent studies, Ouyang et al investigated the mechanisms by which OTUB2 regulates KRT80 stability and thereby promotes proliferation in GC. First, the researchers observed that both OTUB2 and KRT80 proteins were simultaneously overexpressed in GC, although the mRNA level of KRT80 was not as elevated as that of OTUB2. This phenomenon was attributed to OTUB2 regulating KRT80 protein expression through post‐transcriptional regulation. In their study, the proliferative capacities of AGS cells were significantly reduced by OTUB2 and KRT80 knockdown, respectively, using lentivirus technology compared with control cells, and both showed the same degree of effect. In addition, KRT80 supplementation restored growth and proliferation in OTUB2‐knockdown AGS cells, and KRT80 overexpression significantly promoted the cell growth capacity of MKN45 cells.^24^ These results illustrate a relationship between KRT80 and GC cell proliferation, and a specific role for OTUB2 in GC cells is to regulate the expression of KRT80 proteins. As mentioned above, OTUB2 regulates KRT80 protein expression in a post‐transcriptional manner. Specifically, OTUB2 stabilizes the KRT80 protein via deubiquitylation of Lys48 and Lys63 linked, as a result KRT80 is not degraded by the proteasome mediated by ubiquitin.^24^ This conclusion was supported by a ubiquitin pull‐down assay for flag‐tagged KRT80 and a Western blot assay. The researchers also investigated the relationship between OTUB2 and KRT80 and the AKT pathway and found that both OTUB2 and KRT80 can activate the AKT pathway. In conclusion, KRT80 is deubiquitinated by OTUB2, which activates the PI3K/AKT pathway that promotes gastric cancer cell proliferation and growth; KRT80 is essential in this process (Figure 1).^24^ Correlation analysis in 90 GC patients showed that high OTUB2 expression levels were positively correlated with tumor T stage, AJCC stage, and degree of differentiation. Simultaneously, overall survival (OS) was lower in patients with high OTUB2 protein expression than in those with low OTUB2 protein expression.^24^

In the present experiments, the researchers investigated in detail that the regulatory effect of OTUB2 on KRT80 in GC cells was achieved through a post‐translocation mechanism, and thus there was no significant increase in KRT80 mRNA levels. This finding provides a new perspective to study the mechanism of action of KRT80. Because of the close association of OUTB2 with KRT80, it is reasonable to speculate that the high level of KRT80 protein is responsible for the low OS of patients with high OTUB2 protein expression.

In the study by Ishikawa, A. et al, KRT80 was suggested to be potentially associated with the ANXA10 regulatory pathway in GC, and ANXA10 knockdown was able to increase the proliferation^25^, ^39^ and invasiveness^40^ of GC cell lines as well as their sensitivity to 5‐FU.^41^

GC has contributed to much of the research on KRT80 in cancer. These studies suggest that KRT80 plays an essential role in GC and acts through the activation of the PI3K/AKT signaling pathway. However, there may be multiple upstream regulatory genes of KRT80 that interact with each other and collectively affect the expression of KRT80. Although some analysts have suggested that KRT80 may be associated with poor patient prognosis, there is insufficient clinical evidence to support this idea and this aspect needs to be added in future studies.

### 4.3 KRT80 and colorectal carcinoma (CRC)

**FIGURE 2:**
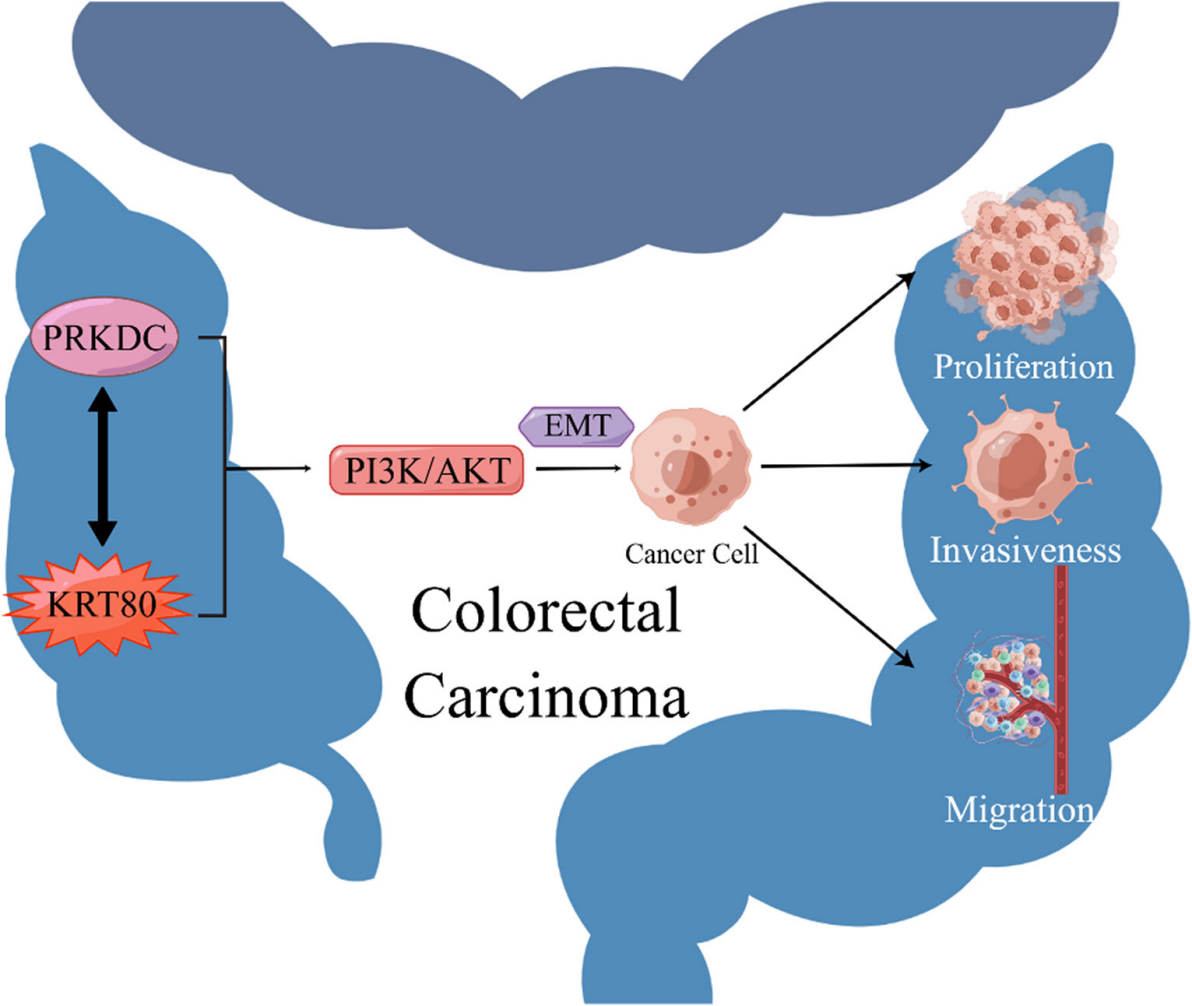
KRT80 interacts with PRKDC to activate the PI3K/AKT pathway and promote EMT in CRC.

In the studies by Li et al and Lin et al, the overexpression of KRT80 in CRC tissues was examined in TCGA and other databases; in addition, using a PCR assay, they both demonstrated that the expression level of KRT80 mRNA was significantly increased in CRC tissues compared to normal tissues. Li et al also determined that KRT80 protein levels were significantly upregulated in CRC tissues using a Western blot assay, and Lin et al determined that KRT80 mRNA expression was significantly increased in CRC cell lines such as HCT116 compared to the normal colorectal cell lines FHC and CCD18CO.^23^, ^26^ A survival analysis of 120 CRC patients suggested that the disease‐free survival (DFS) and OS of the higher KRT80 expression group were much poorer than those of the lower KRT80 expression group, and multivariate analysis showed that KRT80 expression was an independent poor prognostic factor for DFS and OS in CRC patients.^23^ The experiments showed that CRC cells with KRT80 knockdown exhibited decreased migration, invasiveness, viability and proliferation.^23^, ^26^ More importantly, the role of *KRT80* was mediated through interaction with PRKDC, followed by activation of the PI3K/AKT pathway and promotion of epithelial‐mesenchymal transition (EMT) in CRC cells (Figure 2).^23^ It has been shown that EMT plays a crucial role in cancer invasiveness and metastasis.^42^, ^43^, ^44^, ^45^ Overexpression of KRT80 promoted EMT‐mediated changes in cell morphology from round to polygonal and increased the expression of p‐AKT (Ser 473). In contrast, CRC cells expressing EMT‐related proteins were suppressed by KRT80 silencing.^23^ PRKDC is thought to play an important role in AKT activation as a member of the phosphatidylinositol 3‐kinase family.^46^, ^47^, ^48^ The results of Co‐IP assay showed that KRT80 protein could interact with PRKDC protein, and LSCM detected that the colocalization of KRT80 and PRKDC in CRC cells was mainly restricted to the nuclear membrane, proving the interaction between them.^23^ In addition, KRT80 may interact with PPP1CA in CRC cells.^26^

The UALCAN database was used to analyze 274 TCGA colon adenocarcinoma samples. Wu et al found that except for the upregulation of KRT80, its expressions were significantly higher in the later stages (stages III and IV) of CRC than in the earlier stages (stages I and II).^49^ However, KRT80 expression was not significantly associated with the outcome of CRC patients in this study.^49^ More importantly, KRT80 may act primarily through the canonical Wnt signaling pathway, the essential gene of which is CTNNB1 in CRC, as demonstrated by NCI‐Nature enrichment and PPI network analysis.^49^

Through bioinformatics analysis, Jun et al found that KRT80 may act as a hub mRNA of the miRNA‐mRNA network and may play a critical role in CRC development via exosomes.^27^ In addition, KRT80 can distinguish CRC tumors from normal tissues and the phenotype of MSS from MS‐H, which is associated with the tumor microenvironment and the efficacy of immune checkpoint therapy in CRC, making KRT80 a potentially important biomarker for CRC.^27^

KRT80 has been shown to act through the PI3K/AKT pathway. However, theories that it may act on CRC cells through the Wnt pathway and exosomes are still in the bioinformatics stage, and these theories need further experimental validation. As research progresses, the mechanism of action of KRT80 in CRC will be further elucidated and its potential as a biomarker and therapeutic target will increase.

### 4.4 KRT80 and non‐small‐cell lung cancer (NSCLC)

Yuting et al found that the expression of KRT80 in NSCLC tissues was higher than that in adjacent normal tissues of patients, and the overexpression rate of KRT80 in lung adenocarcinoma (LUAD) tissues was significantly higher than that in lung squamous cell carcinoma (LUSC) tissues, based on the immunocytochemistry assays of 176 patients.^29^ However, no correlation was found between KRT80 expression level of NSCLC tissue and clinicopathological factors (sex, age, smoking history, pathology type, lymph node status, T stage or AJCC stage) of patients.^29^ Sanada et al found that patients with LUAD who expressed high levels of KRT80 had a poor prognosis, and KRT80 closely correlated with the molecular pathogenesis of LUAD based on TCGA database analysis.^30^

Existing studies suggest that KRT80 plays a more important role in LUAD than in LUSC, but there is a lack of research on its specific effects and mechanisms on the biological functions of LUAD cells. Given the controversial effects of KRT80 on clinical indicators in LUAD patients, this aspect remains to be explored.

### 4.5 KRT80 and breast cancer (BC)

**FIGURE 3:**
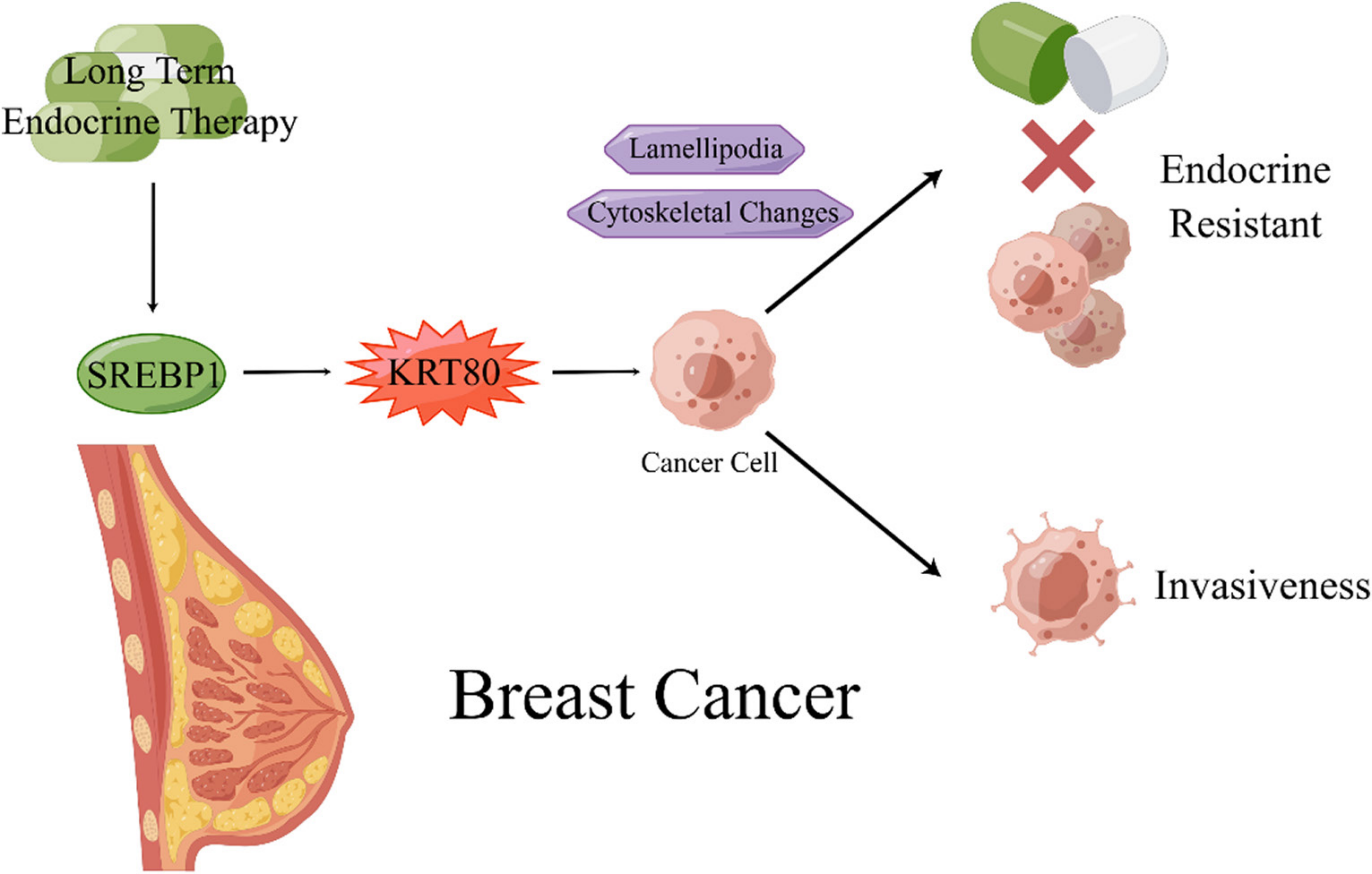
KRT80 is activated by SREBP1 and is involved in the formation of endocrine‐resistant ERα BC.

Perone et al demonstrated that the mechanism of phenotypic reprogramming induced by endocrine therapy can drive behavioral changes in endocrine‐resistant ERα BC and that KRT80 is involved in this process (Figure 3). In endocrine‐resistant BC cells, SREBP1 was aberrantly activated^31^ and bound to core‐E1, which was shown to be a critical KRT80 core enhancer strongly associated with KRT80 transcription and KRT80 overexpression. Overexpression of KRT80 upregulated the expression of cytoskeleton‐related genes, such as SEPT9, and downregulated several genes that play central roles in cancer biology, such as negative regulators of migration and tumor suppressors. All of these changes led to cytoskeletal rearrangements in BC cells. At the same time, a complex actin cytoskeleton is formed in BC cells as a result of KRT80 filament reorganization, which promotes lamellipodia formation. Both cytoskeletal changes and lamellipodia were associated with increased BC cell stiffness in vitro and in vivo, and enhanced the myriad multicellular invasiveness programs known as collective invasiveness of BC cells; i.e., KRT80 overexpression promoted BC cell invasiveness. Cytoskeletal changes and lamellipodia also lead to more extensive, more mature paxillin focal adhesions of BC cells; i.e., KRT80 overexpression promoted BC cell adhesion.^32^

Existing studies have detailed the critical role of KRT80 in BC resistance and the mechanism of action, but the biological functions of KRT80 on BC cells need to be more systematically investigated and elucidated. In addition, as female BC is the most common cancer worldwide,^50^ studies on the prognostic impact of KRT80 on BC patients should also be emphasized.

### 4.6 KRT80 and ovarian cancer (OC)

**FIGURE 4:**
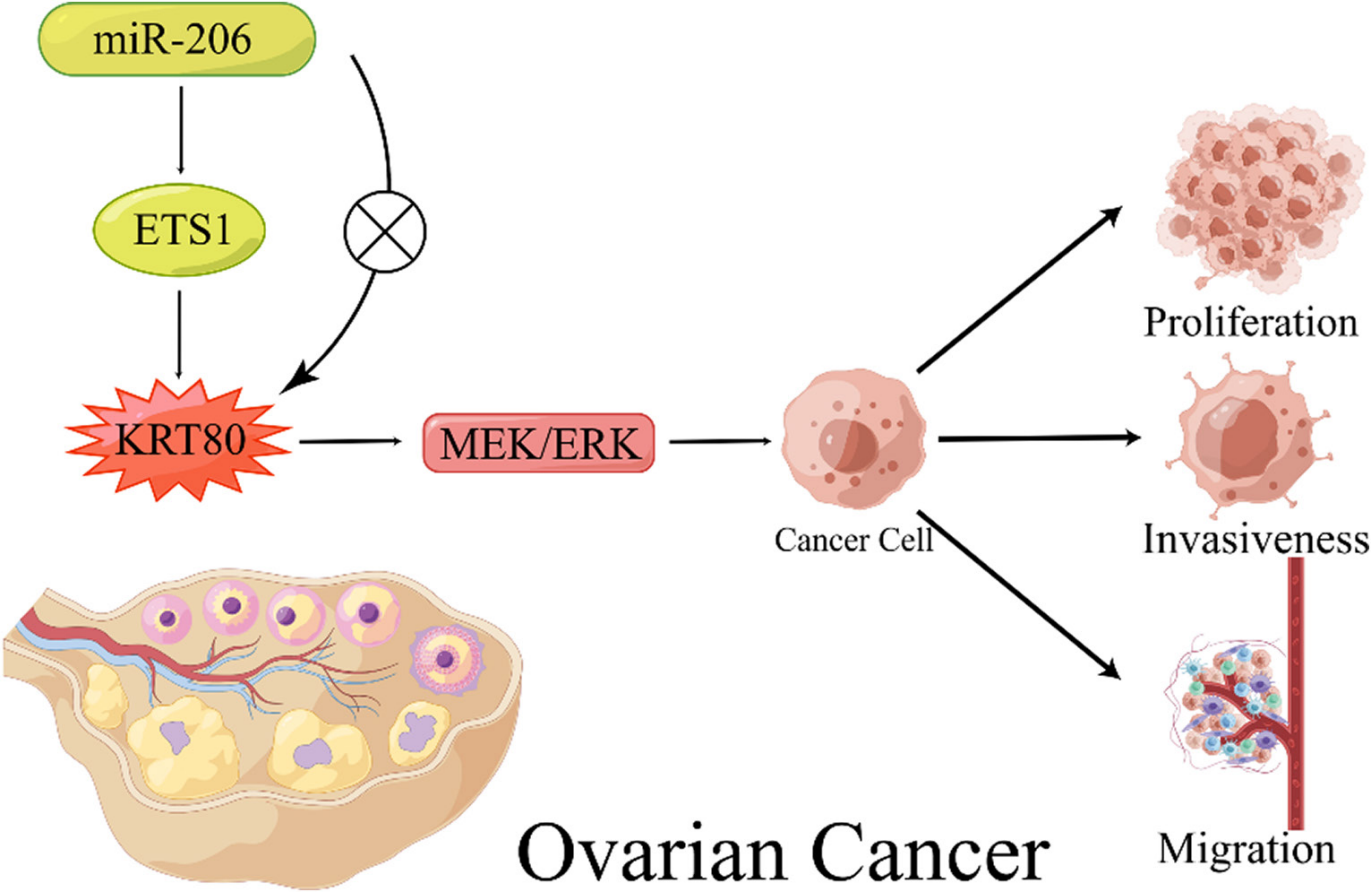
KRT80 affects OC cells through the miR‐206‐ETS1‐KRT80 axis and the MEK/ERK pathway.

Liu et al determined the important role of KRT80 in OC. They found that KRT80 was significantly higher and more positively expressed in the OC group than in the normal and benign groups.^33^ Analyses of databases such as Oncomine have confirmed similar results.^33^ An analysis of 102 OC patients revealed a significant correlation between *KRT80* expression and FIGO stage and lymph node metastasis, i.e., higher *KRT80* expression was correlated with a later FIGO stage and a higher rate of lymph node metastasis.^33^ In addition, they found that patients expressing high levels of KRT80 had a significantly lower five‐year survival rate than those expressing low levels of KRT80 and that KRT80 expression level may be an independent prognostic factor for OC patients.^33^ Furthermore, the researchers confirmed that KRT80 overexpression could significantly increase the proliferation, invasiveness, migration, and EMT of OC cells and enhance the transition of OC cells from G1 to S phase. The opposite effect was observed in the KRT80 knockdown groups, and the cell cycle of OC cells was arrested in the G0/G1 phase.^33^ More importantly, the role of KRT80 is mediated by the miR‐206‐ETS1‐KRT80 axis and the MEK/ERK pathway in OC cells (Figure 4), and ETS1 was confirmed to be a direct target of miR‐206.

In addition, using ChIP and microarray analysis, ETS1 was found to regulate KRT80 expression at the transcriptional level by binding upstream of the KRT80 promoter sequence −616 bp to −486 bp. Pathway enrichment analysis using databases such as GSEA showed that the MAPK pathway was associated with KRT80 and that the MEK/ERK pathway was activated by KRT80, as demonstrated by Western blot assay. Transfection with miR‐206 mimics or inhibitors did not significantly affect KRT80 protein expression levels, indicating that miR‐206 does not directly target KRT80.^33^ The explanation for this phenomenon requires further investigation.

The study of KRT80 in OC has revealed a new downstream regulatory pathway of KRT80, the MEK/ERK pathway, which provides a new perspective to our understanding of this unique gene. As in GC and CRC, there may be more than one pathway in which KRT80 plays a role in OC, and given its specificity, it is of interest to explore the relationship between the MEK/ERK pathway and KRT80 in cancer.

### 4.7 KRT80 and other cancers

In a study by Ulbrich, C. et al, KRT80 expression in follicular thyroid cancer (FTC) cells was significantly upregulated after short‐term microgravity, which may be associated with changes in the distribution and amount of intermediate filaments in FTC cells.^34^ A risk score model established by Chen et al for 12 metabolism‐related genes, including KRT80, could be used as a prognostic marker for clear cell renal cell carcinoma (ccRCC), which has a strong relationship with metabolism, immune infiltration, and immune checkpoints.^35^ Wang et al demonstrated that the potent and systemic pan‐AMPK activator MK8722 could inhibit cancer proliferation, invasiveness and migration of pancreatic cancer cells (PCCs) through multiple pathways. This process could be achieved through the regulation of KRT80 and other genes.^28^ Ji et al found that overexpression of lnc‐TCONS_00049140 could decrease the expression of KRT80 protein and generate a phenotype of increased cell proliferation and increased melanin production by mouse melanocytes, suggesting that the KRT80 gene is involved in tumor suppression in mouse melanoma.^36^

All these studies suggest that KRT80 has great research potential in cancer and is likely to be a new cancer marker and therapeutic target. However, its research in some cancers is superficial, and more systematic and in‐depth studies are warranted to accelerate research progress.

## 5 DISCUSSION

The cytoplasm of most eukaryotic cells is composed of cytoskeletal components, including mainly microfilaments, microtubules, and intermediate filaments.^2^ IFs are highly dynamic and rapidly changing components of the cytoskeleton. They not only form a dense meshwork that provides structural support to cells and organizes the location of organelles, but also extend to the cell periphery to maintain cell and tissue adhesion. Humans possess 54 functional keratins classified into four types, and similar to all other IF proteins, keratin monomers consist of a central α‐helix and variable end domain structures at each end.^51^, ^52^ One type I keratin and one type II keratin form a heterodimer and sequentially assemble to form tetramers, unit filaments, and keratin IF filaments with a thickness of 10 nm.^53^, ^54^, ^55^

KRT80 is a human IF type II epithelial keratin gene located on chromosome 12q13.13, the terminal centromere of the human type II keratin gene domain.^19^ KRT80 is widely expressed in human epithelial cells and pairs with more than 20 different type II keratins to form IF heterodimers in various epithelial cells. The distribution of KRT80‐containing IF in cells is also distinctive. At early stages of differentiation, it is located at the cell edge near desmosome plaques, and in terminally differentiated cells it is distributed throughout the cytoplasm.^21^

The relationship between KRT80 and neoplasms has been studied extensively, particularly in gastrointestinal tumors such as GC. KRT80 has been shown to be highly expressed in a variety of cancers, including ESCC, GC, CRC, NSCLC, OC, and endocrine‐resistant BC. Furthermore, KRT80 has been shown to play an important role in the proliferation, migration and invasiveness of several types of cancer cells, including ESCC, GC, CRC, BC and OC, and to promote the adhesion of BC. KRT80 was found to have higher DNA methylation in grade 2 lung adenocarcinoma.^56^

In clinically relevant studies, KRT80 plays different roles in different tumors. In CRC, the results of Li et al showed that the DFS and OS of the higher KRT80 expression patient group were much worse than those of the lower KRT80 expression group, and KRT80 expression was an independent prognostic factor for poor DFS and OS of CRC patients based on a log‐rank test of 120 CRC patients; however, Wu et al found that KRT80 expression was not significantly associated with clinical outcomes of CRC patients in a survival analysis of 466 samples in the TCGA‐CRC dataset. This difference in the actual clinical impact of KRT80 on prognosis in CRC requires further investigation. In NSCLC, no correlation was found between KRT80 expression in cancer tissues and patients' clinicopathologic factors, including sex, age, smoking history, pathology type, lymph node status, T stage, and AJCC stage. In OC, Liu et al found that higher KRT80 expression was associated with later FIGO stage, higher lymph node metastasis rate and lower 5‐year survival rate, and KRT80 expression was an independent prognostic factor for OC patients. In GC, Ouyang et al found a significant decrease in OS in patients expressing high levels of OTUB2, which was positively correlated with T stage, AJCC stage, and differentiation. Given that OTUB2 promotes gastric cancer growth and proliferation by positively regulating the activation of the PI3K/AKT signaling pathway via KRT80, we believe that KRT80 also has clinical effects similar to OTUB2. To date, the relationships between KRT80 and the clinical prognosis of ESCC and BC remain unclear. Based on the laboratory findings in existing studies, it is likely that KRT80 plays an important role in the clinical prognosis of these cancers. The clinical studies will help us to better understand the great potential of KRT80 as a cancer biomarker and therapeutic target, and provide new ideas for cancer diagnosis and treatment.

**FIGURE 5:**
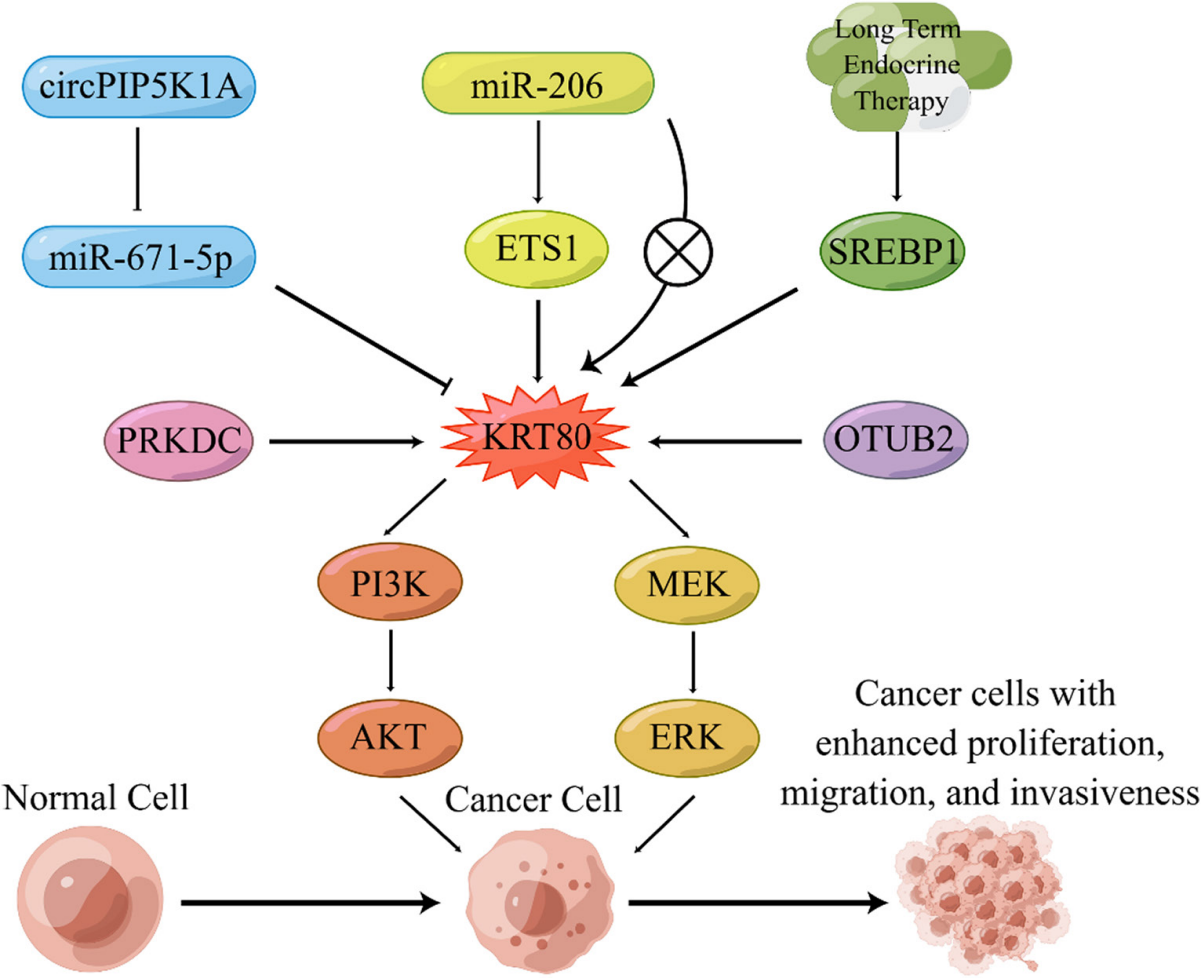
An overall graphical representation on the roles of KRT80 in cancer.

Regarding the mechanism of action of KRT80, several investigators have made positive explorations and some have established relatively detailed pathway models (Figure 5). Song et al found that the role of KRT80 is mediated by the circPIP5K1A‐miR‐671‐5p‐KRT80 axis and the PI3K/AKT pathway in GC cells. Li et al found that KRT80 interacts with PRKDC, followed by activation of the PI3K/AKT pathway and promotion of EMT in CRC cells. Ouyang et al found that KRT80 is deubiquitinated by OTUB2 to activate the PI3K/AKT pathway, which promotes the growth and proliferation of GC cells; KRT80 is essential in this process. Perone et al determined how KRT80 drives the endocrine‐resistant ERα BC behavioral changes induced by endocrine therapy. Liu et al demonstrated the role of KRT80 in the miR‐206‐ETS1‐KRT80 axis and the MEK/ERK pathway of OC cells. In addition, some researchers have suggested possible pathways or interacting factors of KRT80. Wada et al found that the overexpression of KRT80 was probably caused by the downregulation of miR‐143‐3p in ESCC cells. Wu et al and Jun et al suggested that KRT80 may act primarily through the canonical Wnt signaling pathway and may act as a hub mRNA of the miRNA‐mRNA network and may play critical roles via exosomes in CRC cells. We found that PI3K/AKT and MEK/ERK are likely to be the major downstream signaling pathways of KRT80, but it has multiple upstream regulators in different cancers. KRT80 deserves further study in terms of its regulatory pathways, as these findings may shed light and be of great value for future research.

The term “precision medicine” was systematically articulated in 2017, and this new medical paradigm encourages us to go beyond the classic “signs and symptoms” approach to seek more personalized and targeted treatments for different patients.^57^ Molecularly targeted therapies for cancers are a prominent example of this medical model. A frequently cited success example in this regard is the study of the human epidermal growth factor receptor (HER)‐2 gene in breast cancer.^58^ Initially, HER‐2 was found to be an important predictor of disease progression and prognosis in breast cancer patients; subsequently, clinical trials demonstrated the efficacy of the monoclonal antibody trastuzumab, which targets an epitope on the outer structural domain of the HER‐2 protein; today, trastuzumab is widely used in the treatment of HER‐2‐positive breast cancer patients. Existing studies have shown that KRT80 is significantly more highly expressed in a variety of cancer cells than in normal tissues, promotes cancer cell proliferation, invasion and metastasis, and is associated with poorer prognosis in cancer patients. This implies that KRT80 has broad application scenarios in cancer diagnosis, prognosis and treatment. In particular, in molecularly targeted cancer therapy, targeted inhibition of the KRT80 gene, thereby attenuating the proliferation, invasion and metastatic ability of tumor cells and improving patient prognosis, is a promising potential therapeutic approach. Single gene inhibition may not provide satisfactory and significant efficacy, but the combined regulation of multiple molecules or pathways upstream and downstream of KRT80 or the combination of KRT80 inhibitors with other drugs may be an emerging and better therapeutic strategy.

In the future research of KRT80, we should pay attention to both the breadth and depth of research. We should not only validate the aberrant expression of KRT80 in more cancers and its role as a cancer‐promoting oncogene, but also focus on the study of KRT80‐related upstream and downstream regulatory molecules and pathways, for example, there are many gaps that have not been filled in Table 2. The study of molecular mechanisms and pathways is very important because cancer development and progression are often influenced by complex regulatory networks, and these studies can better clarify the position of KRT80 in the regulatory network and also contribute to new molecularly targeted therapeutic strategies for cancer.

## 6 CONCLUSION

KRT80 is a human epithelial IF type II keratin gene that is widely expressed in epithelial cells and pairs with more than 20 different type II keratins to form IF heterodimers in different epithelial cells. In many neoplastic diseases, the high expression status of KRT80 and its role in regulating the biological functions of cancer cells have been well established. KRT80 can effectively enhance the proliferation, invasiveness and migration of cancer cells. However, the effects of KRT80 on prognosis and clinically relevant indices in patients with various cancers have not been extensively studied, and even opposite conclusions have been reached in different studies of the same cancer. Based on this, we should add more clinically relevant studies to clarify the prospect of clinical application of KRT80. Many researchers have made great progress in studying the mechanism of action of KRT80. However, their studies should be extended to more cancers to find common regulators and signaling pathways of KRT80 in different cancers. KRT80 may have far‐reaching effects on the human body, and this marker may play a crucial role in the function of cancer cells and the prognosis of cancer patients, so it has a promising future in the field of neoplasms.

## AUTHOR CONTRIBUTIONS

**Xin‐Yuan Wei:** Conceptualization (equal); data curation (lead); formal analysis (lead); funding acquisition (equal); investigation (equal); methodology (lead); project administration (lead); software (equal); visualization (lead); writing – original draft (lead); writing – review and editing (lead). **Jie Zhao:** Conceptualization (equal); visualization (equal); writing – original draft (equal); writing – review and editing (equal). **Hao‐Bin Tong:** Conceptualization (equal); data curation (equal); funding acquisition (equal); investigation (equal); project administration (equal); resources (equal); software (equal); writing – review and editing (equal). **Shang‐Jie Cheng:** Conceptualization (equal); data curation (equal); investigation (equal); project administration (equal); writing – review and editing (equal). **Na He:** Conceptualization (lead); investigation (lead); methodology (equal); project administration (equal); resources (lead); supervision (lead); validation (lead); writing – original draft (equal); writing – review and editing (equal). **Fei‐Xue Song:** Conceptualization (lead); funding acquisition (lead); methodology (equal); project administration (equal); resources (lead); supervision (lead); validation (lead).

## CONFLICT OF INTEREST STATEMENT

The authors declare that they have no known competing financial interests or personal relationships that could have appeared to influence the work reported in this paper. All figures were drawn by Figdraw.

## ETHICAL APPROVAL STATEMENT

Not applicable.

## CLINICAL TRIAL REGISTRATION NUMBER

Not applicable.

## Data Availability

The data supporting the conclusions of this study are openly available in papers listed in the references.
